# Tracheotomy

**Published:** 1895-02-09

**Authors:** 


### TRACHEOTOMY.

Tracheotomy is often a truly life-saving operation, while in most of the cases in which its performance really comes in question, even if life be not saved, at least the patient is spared the horrors of death by suffocation. All the more important, then, is it that the surgeon should be so well prepared that his interference, if it do no good, at least shall do no harm. Now this preparedness is not attainable exclusively by practice. It only happens to the few to do a large number of tracheotomies; experience is good, but something far different from experience is wanted for the safe performance of this operation, viz, a clear mental picture of the relations of the parts, a crisp conception of the different steps in the operation, and a determination not to be scared away from the details of a well-planned proceeding by accidents which may happen in its course. No one need be deterred from tracheotomy by lack of experience. It not uncommonly happens that quite a junior house surgeon, perhaps as his very first real operation, performs tracheotomy thoroughly well, better than many a senior, and this not from practice or experience, but from his clear anatomical recollections and his determination to go through the operation step by step, as he has been taught to do. What, then, are the steps in this operation?

(*a*) The first step is a mere matter of an incision in the median line, running from the upper border of the cricoid cartilage, or even a little higher, for about an inch and a half downwards. It is essential that this incision should be in the middle line, and that the position of the cricoid should be accurately defined. It is no use feeling for the position of the parts until the head is well thrown back over the sand pillow, for in extension the larynx will move quite the width of a vertebra. The skin and larynx must be steadied by the finger and thumb or the first two fingers of the left hand, while this incision is being made, and it must be remembered that the chest, heaving as it is, must not be used as a support for the right hand. The incision must be carried as deep as may be necessary to open up the interval between the sterno-thyroid muscles. This should be done by a succession of clean cuts; there may be hæmorrhage, but so long as the parts are not displaced, the head is kept exactly straight, and the knife keeps to the middle line, there is no risk. Such bleeding as occurs should be stopped by pressure forceps.

(*b*) Having arrived at this point, although the trachea can be felt under the finger, it is still separated from it by a layer of fascia which runs down from the hyoid bone and encloses the isthmus of the thyroid below. This has now to be opened, and there are several ways of doing so. The simplest plan when there is assistance and good light is, still keeping the larynx steadied by the fingers of the left hand, to divide this fascia cleanly in the middle line. In the cellular tissue underneath probably veins will be seen, and also the isthmus of the thyroid, the first of which may be drawn aside, and the latter downwards with the blunt hook. Many surgeons, however, dislike the use of the knife in these deeper parts. The deep fascia may then, if preferred, be split by a raspitory from the upper limit of the incision down to the isthmus, and by means of a probe and blunt hook the veins and the isthmus may be drawn away.

Another plan is to tear the parts asunder by means of two pair of forceps, one in each hand, working down steadily until the trachea is clean; or a transverse incision may be made in the fascia on a level with the cricoid cartilage, and the tissues be separated from the trachea with a probe, and pulled down by a blunt hook; or lastly, a small transverse opening in the fascia being made over the cricoid, the blades of two pressure forceps may be carried down in front of the trachea, one on each side of the middle line, each forceps taking in its bite most of the tissues between the trachea and the bottom of the wound already made. These tissues can then be divided in the middle line between the forceps, and by separating the latter the trachea will be left bare with very little hæmorrhage.

(*c*) Having now reached the trachea, the next thing is to incise it. In doing this the use of a sharp hook to steady the trachea is of the greatest assistance. Not only does it give a fixed point to aim at, but it much facilitates the introduction of the tube afterwards. The hook is to be fixed into the substance of the cricoid cartilage, which is then to be slightly drawn up; the knife is then made to stab the trachea and cut up towards the hook. The knife is now to be laid down, and if a considerable quantity of mucus happens to have been blown out into the wound it may be wiped away; but whatever happens, and however much the child may cough and struggle, the hook must not be removed. One side of the incision in the trachea is now caught by a pair of forceps and the wound is held open while (*d*) the tube is introduced. Herein comes the great use of the hook. It is impossible to lose the opening if one knows its relation to the position of the hook, and, even if the parts are obscured by froth and mucus, drawing the point of the forceps across the track of the incision will always open it; and it should be noted that once the trachea is held open by forceps there need be no hurry about the introduction of the tube, which may be done quietly after the first spasmodic coughing is got over.

A word must be said about antiseptics. It is clearly impossible entirely to protect such a wound, but free swabbing with a lotion, such as carbolic lotion, which coagulates the tissues, is probably of considerable service in preventing the occurrence of that deep cellulitis which sometimes occurs and is a source of great danger after the operation. An anæsthetic should always be given.

